# Clinical characteristics, diagnosis, treatment and outcomes of patients living with HIV and co-infected with tuberculosis and histoplasmosis: a 5-y retrospective case series

**DOI:** 10.1093/trstmh/trad104

**Published:** 2024-01-27

**Authors:** María Eugenia Castellanos Reynosa, Maria Eugenia Caal, Danicela Mercado, Narda Medina, Juan Carlos Pérez, Theophilus I Emeto, Eduardo Arathoon

**Affiliations:** Public Health and Tropical Medicine, College of Public Health, Medical and Veterinary Sciences, James Cook University, Townsville, Queensland, Australia; Asociación de Salud Integral, Guatemala City, Guatemala; Clínica Familiar Luis Ángel García, Hospital General San Juan de Dios, Guatemala City, Guatemala; Asociación de Salud Integral, Guatemala City, Guatemala; Asociación de Salud Integral, Guatemala City, Guatemala; Public Health and Tropical Medicine, College of Public Health, Medical and Veterinary Sciences, James Cook University, Townsville, Queensland, Australia; Asociación de Salud Integral, Guatemala City, Guatemala; Clínica Familiar Luis Ángel García, Hospital General San Juan de Dios, Guatemala City, Guatemala

**Keywords:** AIDS, co-infection, epidemiology, histoplasmosis, HIV infection, tuberculosis

## Abstract

**Background:**

In Latin America, tuberculosis (TB) and histoplasmosis are two of the most frequent opportunistic infections affecting people living with human immunodeficiency virus (HIV). However, there are limited data on the clinical characteristics and outcomes of patients with concurrent TB and histoplasmosis infections.

**Methods:**

This was a retrospective observational study to describe the clinical, epidemiological and laboratory characteristics and outcomes of 21 patients living with HIV (PLHIV) who were diagnosed with concurrent histoplasmosis and TB between 2017 and 2021 in Guatemala City, Guatemala.

**Results:**

Most patients were male and were newly diagnosed with HIV. All patients had advanced HIV disease (AHD). They presented with a median CD4 count of 20 cells/µl. The most common symptoms reported by the patients were fever, weight loss, cough and diarrhoea. Twelve patients died within 6 months of baseline evaluation, for a mortality rate of 57.1%.

**Conclusions:**

PLHIV with concurrent TB and histoplasmosis infections are characterised by AHD, predominantly presenting with disseminated forms of these infections and with unspecific symptoms and signs. This evidence calls for early HIV and opportunistic infection screening and insights into the challenges and opportunities for the efficient diagnostic and therapeutic management of patients with AHD with concurrent histoplasmosis and TB infections.

## Introduction

Human immunodeficiency virus (HIV) infection and advanced HIV disease (AHD) increase the risk of opportunistic infections. Among these, tuberculosis (TB) and histoplasmosis are two of the most common acquired conditions. TB is a bacterial infection caused by *Mycobacterium tuberculosis*, which resulted in 1.6 million deaths globally in 2021, with 187 000 of them occurring in people living with HIV (PLHIV).^1^ Histoplasmosis is a fungal infection caused by *Histoplasma capsulatum*, which is widely distributed but mainly reported in the Americas. Histoplasmosis can manifest as an asymptomatic or disseminated infection. PLHIV can present with symptomatic and disseminated disease.^2,3^

Despite efforts to improve the diagnosis and management of TB in PLHIV, histoplasmosis remains, in most areas, a neglected condition due to the lack of epidemiological data on its actual burden.^4,5^ A recent modelling study estimated that the proportion of the population previously exposed to *H. capsulatum* is 32.2%, and the incidence of histoplasmosis in PLHIV is 1.48 cases per 100 population.^6^ The study also suggested that histoplasmosis could be as important or more critical than TB as a cause of acquired immunodeficiency syndrome (AIDS)-related deaths in Latin America.^6^

Histoplasmosis infection in PLHIV is often misdiagnosed as TB, leading to poor outcomes.^7^ Additionally, a previous study in Guatemala revealed that among newly diagnosed PLHIV with opportunistic infections, 8.1% experience multiple co-infections and, of these, 32.3% have histoplasmosis/TB co-infections.^8^ However, there are limited data on the clinical characteristics and outcomes of PLHIV with concurrent TB and histoplasmosis infections. A case series study by Agudelo *et al.*^9^ described the clinical features of 14 PLHIV with concomitant TB and histoplasmosis recorded over 19 years (1992–2011). However, this study was conducted more than a decade ago, and significant changes have occurred in the diagnosis and treatment of these infections since then.

Therefore, more research is needed to understand the impact of concurrent TB and histoplasmosis infections on PLHIV, their clinical and laboratory characteristics and the optimal therapeutic strategies. In this study we aimed to comprehensively describe the epidemiological, clinical and laboratory features, treatment and outcomes of PLHIV diagnosed with concurrent TB and histoplasmosis.

## Methods

### Study setting

The study was conducted at Clínica Familiar Luis Ángel García (CFLAG), a large HIV referral centre in Guatemala City, Guatemala, affiliated with the National Hospital General San Juan de Dios. In Guatemala, the estimated prevalence of HIV among adults aged 15–49 years is 0.2%.^10^

The CFLAG serves approximately 4500 PHLIV per year, both hospitalised and walk-in patients.^11,12^ The CFLAG follows the national HIV testing algorithm for the diagnosis of HIV.^13^ Prenatal/obstetric patients universally undergo HIV testing as part of routine care. For all other patients, HIV testing is either self-selected or initiated through the healthcare provider. Importantly, the testing services are provided free of charge and individuals diagnosed with HIV receive rapid initiation of antiretroviral therapy (ART) within 7 d unless contraindications exist. In 2017, the CFLAG implemented a screening program for opportunistic infections among PLHIV and reported that 51.2% of newly diagnosed HIV patients had AHD, with an overall opportunistic infection incidence of 21%.^8^

### Study design

We performed a retrospective case series of PLHIV who were diagnosed with concurrent histoplasmosis and TB at the CFLAG between 2017 and 2021. We aimed to describe the clinical, epidemiological and laboratory characteristics and outcomes of these patients.

### Study population and inclusion criteria

We included patients ≥18 years of age who were living with HIV and who were enrolled in the screening program for opportunistic infections at the CFLAG between 2017 and 2021. The inclusion criteria were laboratory confirmation of histoplasmosis and TB within a 4-month interval and the availability of medical, laboratory and pathology records for data extraction. We excluded patients who had incomplete or missing records.

### Data collection and variables

We developed a standardised data collection tool to extract relevant information from medical records. We used Excel software (Microsoft, Redmond, WA, USA) to create a database with cloud backup using a secure online server. A trained researcher collected the data by reviewing the records and entering them into the database.

The screening date for opportunistic infections was considered as the baseline evaluation. Variables collected from this baseline evaluation included age, gender, occupation, residence area, time living with HIV, CD4 count, HIV viral load, signs and symptoms, clinical laboratory data, laboratory diagnosis method for histoplasmosis and TB, diagnosis of other opportunistic infections and date of screening. We also collected data on the treatment for histoplasmosis and TB, follow-up CD4 counts, medical evaluations and vital status after 6 months.

### Laboratory diagnosis methods for TB and histoplasmosis

During the study period, the CFLAG has used various laboratory techniques for diagnosing histoplasmosis and TB.^14^ We collected data from the following laboratory methods: in-house polymerase chain reaction (PCR) for detection of *M. tuberculosis* and *H. capsulatum*, culture of biological samples for *Mycobacterium* sp. using Lowenstein–Jensen medium and/or *Mycobacterium* growth indicator tubes (Becton Dickinson, Cockeysville, MD, USA), culture of biological samples for *Histoplasma* sp. using Sabouraud dextrose agar 4% and Mycosel Agar (Becton Dickinson), detection of urine *H. capsulatum* antigen (Histoplasma GM enzyme immunoassay, Immuno-Mycologics, Norman, OK, USA), lateral flow antigen lipoarabinomannan assay (LF-LAM) for TB (Determine TB-LAM, Abbott, Waltham, MA, USA), blood culture for *Histoplasma* sp. using Isolator tubes (Du Pont, Wilmington, DE, USA) followed by inoculation on fungal media and GeneXpert platform for TB (Cepheid, Sunnyvale, CA, USA). In one case we used a referral laboratory diagnosis of TB to confirm the infection.

### Outcome measures

The primary outcome measure was mortality after 6 months from the date of the baseline evaluation. We obtained the date of death from medical records or reports from family members. We classified deaths into two categories based on the time of death: early death (death within 30 d from baseline evaluation) and late death (death after 30 d from baseline evaluation).

### Analytical strategy

We summarised categorical data as counts and percentages. We analysed numerical data using appropriate measures of central tendency and dispersion. We also calculated the time from baseline evaluation to ART initiation. For patients who started ART within a few days before the baseline evaluation, we assumed that they initiated ART on the same day as the baseline evaluation.

We reported the proportion of patients with concurrent histoplasmosis/TB who died after 6 months from baseline evaluation. Based on the exploratory analyses, we created two categories of death according to the time of death: early death (death ≤30 d) and late death (death >30 d).

We explored crude associations between therapy received (ART, antifungal, anti-TB) and 6-month mortality using Fisher's exact test.

We performed all analyses using R version 3.6.2 (R Foundation for Statistical Computing, Vienna, Austria).^15^ The package gtsummary was used to create and report the descriptive results and summary tables.^16^

## Results

### Sociodemographic characteristics of the patients with concurrent co-infections

We identified 26 patients with HIV, TB and histoplasmosis between January 2017 and December 2021, of whom 21 met the inclusion criteria. At baseline evaluation, most patients were male (86%), heterosexual (81%) and had a median age of 37 years (interquartile range [IQR] 27–41) (Table 1). Most patients (62%) were newly diagnosed with HIV, while seven patients had discontinued ART for at least 90 d and were returning to care. One patient was on ART but had symptoms of opportunistic infections.

**Table 1.** Sociodemographic baseline data of 21 PLHIV with histoplasmosis and TB co-infection.

Characteristics	Values
Sex, n (%)	
Female	3 (14)
Male	18 (86)
Age (years), median (IQR)	37 (27–41)
HIV diagnosis at the time of screening, n (%)	
Yes	13 (62)
No	8 (38)
Receiving ART treatment, n (%)	
Yes	1 (5)
No	20 (95)
Sexual orientation, n (%)	
Heterosexual	17 (81)
Other	4 (19)
Education, n (%)	
None/primary	13 (62)
Secondary	7 (33)
University	1 (4.8)
Employment status^a^, n (%)	
Employed	11 (55)
Housewife	3 (15)
Unemployed	6 (30)

a Data for 20 patients.

### Clinical presentation

The patients had different manifestations of TB and histoplasmosis. Eleven patients had extrapulmonary TB and disseminated histoplasmosis, eight had pulmonary TB and disseminated histoplasmosis and two had pulmonary co-infections. Among the patients with extrapulmonary TB, the most common form was disseminated TB (n=8), followed by tuberculous lymphadenitis (n=1) and tuberculous meningitis (n=2).

**Figure 1.**
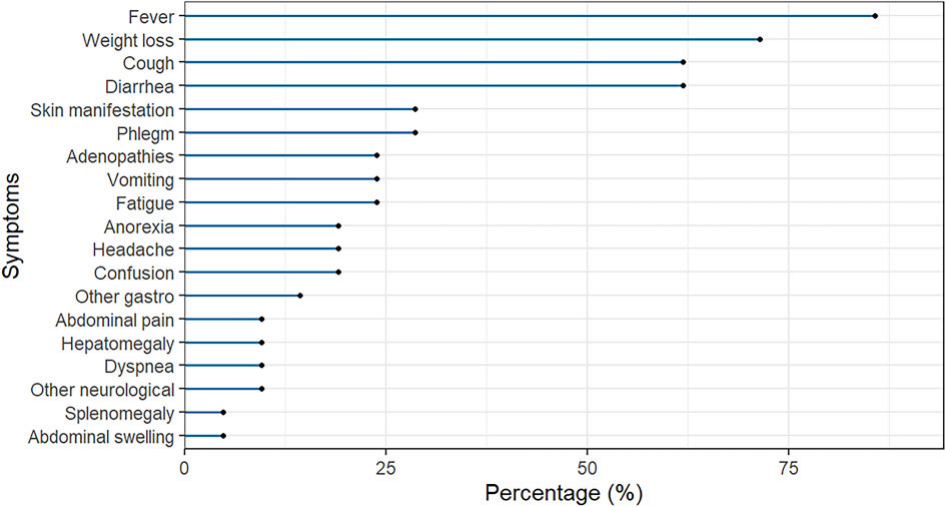
Frequency of symptoms reported by 21 PLHIV and with both histoplasmosis and TB co-infection attending the CFLAG, Guatemala City, Guatemala, 2017–2021.

The median body mass index of the patients at baseline evaluation was 19.43 kg/m^2^ (IQR 18.65–21.92). The most common symptoms reported by the patients were fever (86%), weight loss (71%), cough (62%) and diarrhoea (62%) (Figure 1).

Six patients (29%) had skin lesions. These included plaques with crust on the neck or genital area (n=2), pustules on the lower limbs (n=1), hyperpigmented macules on the chest and abdomen (n=1), violaceous papules (n=1) and perianal verrucous lesions (n=1). All these patients had disseminated histoplasmosis.

Three patients (14%) had other opportunistic infections besides TB and histoplasmosis. These were *Pneumocystis jirovecii* pneumonia (n=1), cerebral toxoplasmosis (n=1) and pulmonary coccidioidomycosis (n=1). We tested all patients for cryptococcosis using a cryptococcal antigen lateral flow assay, but none was positive.

We obtained chest X-rays from nine patients and seven of them (78%) had abnormal findings. The most frequent patterns were reticulonodular (n=3), micronodular (n=2) and reticular with microcalcification (n=1).

### Laboratory data

Table 2 summarises the baseline laboratory data of the patients. All patients had advanced HIV disease (CD4 <200 cells/µl), with a median CD4 count of 20 cells/µl (IQR 8–47) and a median HIV viral load of 438 560 copies/ml (IQR 84 716–992 079). Anaemia was the most common laboratory abnormality, affecting 20 of 21 patients (median haemoglobin 10.0 g/dl [IQR 8.20–11.80]). Most patients also had elevated levels of lactate dehydrogenase (80%; median value 447 IU/l [IQR 262–763]) and gamma-glutamyl transferase (75%; median value 157 IU/l [IQR 80–293]).

**Table 2.** Baseline laboratory data for 21 PLHIV with histoplasmosis and TB co-infection.

Characteristics	Values
CD4 count (cells/μl), median (IQR)	20 (8–47)
HIV viral load (copies/ml)^a^, median (IQR)	438 560 (84 716–992 079)
Anaemia^b^	20 (95)
Leukopenia (<10.5×10^9^/μl)	9 (43)
Thrombocytopenia (<150×10^9^/μl)	8 (38)
Increased alanine aminotransferase (>45 IU/l)^a^	11 (55)
Increased aspartate aminotransferase (>40 IU/l)^a^	14 (70)
Increased creatinine (>1.10 mg/dl)^a^	5 (25)
Increased lactate dehydrogenase (>248 IU/l)^a^	16 (80)
Increased alkaline phosphatase (>270 IU/l)^a^	14 (70)
Increased gamma-glutamyl transferase (>71 IU/l)^a^	15 (75)
Hypoproteinaemia (<6 g/l)^c^	6 (32)

Values presented as n (%) unless stated otherwise.

a Data for 20 patients.

^b^Anaemia: haemoglobin <13 g/dl for males and <12 g/dl for females.

^c^Data for 19 patients.

### Laboratory diagnosis of TB and histoplasmosis

Most patients (n=17 [81%]) received a diagnosis of both infections within 2 weeks of diagnosis of the first infection. Different methods were used to diagnose TB and histoplasmosis. Table 3 summarises the results of these methods. For the TB diagnosis, PCR or culture was mainly used. Of these two methods, the positivity rate was higher with PCR (61% vs 29%), particularly among pulmonary samples (80%) (Table 3). Only eight patients were tested with Determine TB-LAM, with six (75%) being positive using this method.

**Table 3.** Laboratory method of diagnosis for 21 PLHIV with histoplasmosis and TB co-infection.

	Patients positive/tested (% positive)		
Disease	Overall (N=21)	Non-pulmonary (n=11)	Pulmonary (n=10)
TB diagnosis			
PCR	11/18 (61)	3/8(38)	8/10 (80)
LF-LAM	6/8 (75)	6/6 (100)	0/2 (0)
GeneXpert	6/11 (55)	2/4 (50)	4/7 (57)
Culture	5/17 (29)	2/9 (22)	3/8 (38)
Referral diagnosis	1/1 (100)	0/0	1/1 (100)
Histoplasmosis diagnosis	Overall (N=21)	Disseminated (n=19)	Pulmonary (n=2)
UAg	15/21 (71)	15/19 (79)	0/2 (0)
Blood culture	2/11 (18)	2/10 (20)	0/1 (0)
PCR	11/19 (58)	9/17 (53)	2/2 (100)
Solid culture	2/17 (12)	2/16 (12)	0/1 (0)

UAg: Histoplasma urine antigen

To diagnose histoplasmosis, all patients were tested with histoplasma urine antigen, and 15 (71%) were positive using this technique. Nineteen patients were evaluated using PCR, and 11 were positive (58%). For the two patients diagnosed with pulmonary histoplasmosis, PCR was the sole method of diagnosis. The blood culture and solid culture positivity rates (18% and 12%, respectively) were low.

### Management and survival status

The 6-month mortality rate among the 21 patients was 57.1% (n=12). Five patients (23.8%) died within 30 d of the baseline evaluation (median survival time 15 d [IQR 13–18]). The remaining seven patients (33.3%) died after 30 d of the baseline evaluation, with a median survival time of 119 d (IQR 110–141).

Sixteen patients (76%) received ART (Table 4). Nine of them started ART within 14 d of their baseline evaluation (range 0–13 d), and of these, six survived (67%). The other seven patients started ART after >14 d after of their baseline evaluation (range 16–89), and of these, three survived (43%). Overall, all survivors received ART within 18 d of their baseline evaluation. In three survivors, temporary ART suspension was instructed by the clinician (33.3%) but resumed within a month. The proportion of patients who received ART was higher among patients who survived (100%) than among those who died (58%, p=0.045).

**Table 4.** Therapy and 6-month outcomes for 21 PLHIV with histoplasmosis and TB co-infection.

Characteristics	Total (N=21)	Dead (n=12)	Survivors (n=9)	p-Value^a^
Received ART	16/21(76)	7/12 (58)	9/9 (100)	0.045
Received antifungal therapy	18/21(86)	9/12 (75)	9/9 (100)	0.2
Received amphotericin B as induction therapy	16/21 (76)	8/12 (67)	8/9 (89)	0.3
Received TB treatment	19/21 (90)	10/12 (83)	9/9 (100)	0.5
Initiation phase was completed	13/21 (62)	4/12 (33)	9/9 (100)	0.005
Continuation phase was completed	9/21 (43)	0/12 (0)	9/9 (100)	<0.001

Values presented as n/N (%).

a Fisher's exact test.

Supplementary Table S1 shows the details of TB and histoplasmosis management and the outcome of each participant. Eighteen participants (86%) received antifungal therapy (Table 4). Three patients died without antifungal treatment.

Among the 19 patients with disseminated histoplasmosis, 16 received amphotericin B as induction therapy. Fifteen of them received amphotericin B deoxycholate (DAmB) at a dose of 0.7 mg/kg/d and one received DAmB plus liposomal amphotericin B (LAmB) at a dose of 1 mg/kg/d. Nine patients received a 14-d amphotericin B treatment course, one received a 13-d treatment course, three patients received longer treatment (range 19–21 d) and three patients died during the amphotericin B induction therapy. One of the patients who received longer treatment was also being treated for coccidiomycosis. Twelve of the 13 patients who completed the induction therapy were then switched to itraconazole as maintenance therapy. Ten of these patients received itraconazole in a dose of 200 mg three times/d, with only one patient switching temporarily to a lower dosage (200 mg twice daily) before being switched back to the higher dosage. For the other nine patients, four stayed on the 200 mg three times/d regimen until the end of the follow-up period or death and five were switched to a lower dosage only after an extended period on the higher dosage (range 141–271 d). Eight of the 12 patients treated with amphotericin B and itraconazole survived (67%).

Three patients with disseminated histoplasmosis did not receive amphotericin B as induction therapy. One received itraconazole at a dose of 200 mg three times/d (as an induction and maintenance therapy) and survived. The other two did not receive any antifungal treatment and died.

There were two patients with pulmonary histoplasmosis, and both died. One was treated with itraconazole at a dose of 200 mg three times/d until death. The other died early without receiving any antifungal treatment.

Anti-TB treatment consisted of an initial phase of 2 months of isoniazid, rifampicin, pyrazinamide and ethambutol, followed by a continuation phase of 4 months of isoniazid and rifampicin. Nineteen patients received this treatment. Two patients died before starting anti-TB treatment. Thirteen patients completed the initial phase (50 doses according to Guatemalan Ministry of Health guidelines). Nine of them received the continuation phase (105 doses). All nine survivors (100%) completed both phases, whereas only 33% of patients who died complete the initiation phase and none completed the continuation phase (Table 4).

There were 16 patients who received ART and anti-TB treatment. Eight patients were initiated on ART within the first 2 weeks of initiating TB treatment and five of them survived (62%). Four other patients were initiated on ART after the first 2 weeks of initiating TB treatment and one of them survived. The remaining four patients were already on ART when the TB treatment was initiated and three survived.

Follow-up CD4 counts within the first 6 months after the baseline evaluation were available for eight survivors. The median time for these measurements was 53 d (IQR 42–72). The median CD4 count among these survivors was 95 cells/µl (IQR 68–170), which represents a median increase of 78 cells/µl (IQR 40–104) from baseline.

## Discussion

This retrospective observational study described the epidemiological, clinical and laboratory characteristics, treatment and outcomes of PLHIV diagnosed with concurrent TB and histoplasmosis in Guatemala.

The results revealed these patients’ presentations and the challenges for their adequate management. The patients had very low CD4 counts at their baseline evaluation (median 20 cells/µl [IQR 8–47]). All the patients had AHD, leading to a higher risk of opportunistic infections and mortality.^17^ In this study, 62% of the patients were diagnosed with HIV during their baseline evaluation. A previous study in Guatemala reported that 81% of the patients presented with an AIDS-defining illness at the time of HIV diagnosis and that most (82%) did not seek care 2 years before the HIV diagnosis.^18^ The finding of a low CD4 count in this study is worrisome, as previous research has shown that a CD4 count of <50 cells/µl, compared with a CD4 count of >500 cells/µl, increased the risk of attrition (patients who died or were lost to follow-up) by 91% (95% confidence interval [CI] 1.63 to 2.24, p<0.001), whereas a CD4 count of 350–499 cells/µl reduced the risk by 21% (95% CI 0.65 to 0.97, p<0.023).^19^

The sociodemographic and clinical features of these patients were compared with those reported by Agudelo et al.,^9^ who conducted a study from 1992 to 2011 in Colombia in PLHIV. Some striking similarities were found. Both populations were predominantly male (86%) and had a similar age (median age 37 years in our study vs 36.5 years in the Colombian study). The most frequent signs and symptoms were unspecific, such as weight loss, fever and cough. A noticeable difference was the lower frequency of diarrhoea reported in the Agudelo et al.^9^ study (21%) compared with this study (62%). Diarrhoea and abdominal pain are common symptoms of gastrointestinal histoplasmosis, likely a disseminated disease manifestation.^20^ In this study, 19 of the 21 patients had disseminated histoplasmosis, which could explain this discrepancy. In the Colombian study, hepatomegaly was detected in 69.2% of the patients and splenomegaly in 54%, whereas in this study <25% of the patients had these findings. A significant difference is that the Agudelo et al.^9^ study used abdominal ultrasound to detect these abnormalities, whereas this study relied only on medical evaluation. A previous work from French Guiana showed that among PLHIV with disseminated histoplasmosis, the prevalence of hepatomegaly (diagnosed by signs and symptoms) was 28%.^21^

The diagnostic accuracy of the laboratory tests varied greatly. *Histoplasma* urine antigen and LF-LAM assay had the highest positivity rate for the extrapulmonary forms of the disease. These findings are consistent with the World Health Organization (WHO) recommendations to use LF-LAM to detect active TB in PLHIV with a CD4 cell count of <100 cells/µl and to use *Histoplasma* antigen to detect disseminated histoplasmosis in PLHIV.^22,23^ PCR was the most sensitive method among the samples tested for pulmonary forms of the disease. Traditional methods like culture had low detection rates. However, as previously reported, no single technique could detect all the cases,^14^ confirming the difficulty of diagnosing these infections separately or concurrently in PLHIV.^24^ Moreover, in low-resource settings, accurate and affordable point-of-care tests in primary care should be available in order to diagnose these infections.

The biochemical profile of this study population showed a high proportion of anaemia, increased lactate dehydrogenase levels and increased gamma-glutamyl transferase levels. A previous study from French Guiana compared laboratory data from 99 patients with TB and 106 with histoplasmosis and found these three measurements were more common in patients with histoplasmosis than in patients with TB.^25^ Therefore, a biochemical profile like this study might lead clinicians to diagnose histoplasmosis exclusively. However, these findings indicate that a comprehensive standardised screening algorithm to detect opportunistic infections and others should be followed in all patients with advanced HIV diagnoses, regardless of their clinical results or biochemical data.^26^

However, more needs to be done for patients with concurrent infections. Little is known about the best treatment for patients co-infected with TB and histoplasmosis. No specific clinical guidelines exist for PLHIV and co-infected with TB and histoplasmosis. These are urgently needed as rifampicin interacts with itraconazole, reducing its levels.^27^ In 2020, the WHO published the first guidelines for detecting and managing disseminated histoplasmosis in PLHIV.^22^ There were four recommendations. Three were related to the treatment of disseminated histoplasmosis: use of liposomal amphotericin B as induction therapy, use of itraconazole as maintenance therapy and prompt ART. The only recommendation addressing co-infection was ‘People living with HIV with TB and histoplasmosis co-infection should receive TB therapy according to WHO treatment guidelines’.^22^

The WHO guidelines recommend that TB patients living with HIV should receive ART within the first 2 weeks of initiating TB treatment.^28^ In this study, five of the eight patients who followed these guidelines survived, whereas only one of the four patients who initiated ART after the 2-week period survived. Delay in ART initiation might have been related to the clinician's consideration to reduce the risk of developing immune reconstitution inflammatory syndrome (IRIS).^29^ A recent meta-analysis showed that in PLHIV with TB and with low CD4 cells (<50 cells/µl), early ART (≤4 weeks) is associated with a higher risk of IRIS but also decreased mortality compared with delayed ART (>4 weeks).^29^ This shows the complex balance that clinicians need to consider when managing these patients, as they are the ones at greater risk of IRIS but also the ones who will benefit most from early initiation on ART.

Most patients with disseminated histoplasmosis received DAmB as an induction therapy. Although DAmB is an alternative induction therapy for disseminated histoplasmosis,^22^ LAmB is considered the preferred choice, as it has lower rates of nephrotoxicity and a higher clinical success rate than DAmB.^30^ However, in many developing countries, such as Guatemala, routine access to this drug is limited due to its high cost.^7,31^ A randomised phase 2 trial in Brazil showed that a single dose of 10 mg/kg of LAmB might produce results similar to the standard treatment of 2 weeks, which might reduce the cost of this treatment.^32^ Global initiatives to increase access to medicines in resource-limited settings should be a priority across all nations.^33^ Only through a comprehensive package for the treatment of HIV and HIV-related conditions such as opportunistic infections will it be possible to adequately manage PLHIV.

The maintenance therapy was itraconazole, but most patients received 200 mg three times/d instead of the recommended guideline of 200 mg twice/d. In Guatemala, there is no availability to monitor itraconazole levels, and there is no availability of rifabutin, which has lower drug–drug interactions than rifampicin, particularly in PLHIV.^34^ Therefore this modification might have been made due to the potential interactions of itraconazole with rifampicin.^35,36^ To ensure that patients receive the appropriate dose of itraconazole, urgent action is needed to measure serum levels of this metabolite after the first 2 weeks of therapy.^37^

In this study, the completion of anti-TB treatment, especially in the intensive phase of treatment, was inadequate. It has been shown that several factors, including socio-economic conditions, individual beliefs, family and health professional support and concurrent treatments, may influence adherence levels in patients with HIV and TB.^38^ Further research is needed to identify more cost-effective interventions to support these patients. Successful anti-TB treatment should involve coordination between public health authorities, clinicians and the patient. Alternative therapies for rifampicin, including other rifamycins, should also be explored in these patients. Newer fluoroquinolones, such as moxifloxacin instead or rifampicin, may be good options, but more work is needed to determine optimal doses and potential treatment failure due to pre-existing resistance to these drugs.^9,39^

We had previously shown that a care package integrating a rapid screening program decreases the mortality of opportunistic infections by 7%.^8^ In this study, this comprehensive diagnostic approach allowed the recognition of concurrent TB and histoplasmosis cases as well as other infections. Without this, many cases might only be recognised as isolated opportunistic infections. Nevertheless, our findings showed a 6-month mortality of 57%, despite the utilization of rapid diagnostic tests as well as the administration of ART, antifungal and anti-TB treatments in adherence with clinical guidelines. This trend underscores the significance of not solely relying on cutting-edge diagnostic and therapeutic methods to reduce mortality rates, particularly when patients present with advanced HIV stages, as observed in this study. Therefore, an expansion of HIV testing is urgently needed,^40^ as well as an increase in community education about HIV and a focus on reducing the stigma and discrimination of this disease in the Latin American region.^41^ Alternative strategies such as HIV self-testing with digital support should be explored, as recent evidence indicates this can be a feasible and acceptable method to increase HIV testing.^42^ These approaches are needed for Guatemala to achieve the 95–95–95 UNAIDS Initiative, which includes that 95% of PLHIV know their serological status.^43^ It is worth emphasizing that overall mortality improvements can be achieved when early HIV diagnosis is combined with screening for opportunistic infections.

This study has significant limitations. First, the small sample size limited the ability to explore mortality-related factors. Second, information bias is a concern, as this was a retrospective study.^44^ However, a medical doctor from the research team reviewed the electronic and clinical records using a standardised collection tool to minimise this bias. Third, the ‘in-house’ PCR used for the molecular diagnosis of TB and histoplasmosis has not been validated, which might compromise the reliability of the results. Finally, this study represents only one centre, which may not represent other settings in Latin America. Nevertheless, this centre is one of the largest healthcare facilities in Guatemala.

In conclusion, people living with HIV who have concurrent TB and histoplasmosis infections are characterised by AHD, predominantly presenting with disseminated forms of these infections and with unspecific symptoms and signs. Early HIV and opportunistic infection screening and access to recommended treatments are needed to prevent progression to AHD and to reduce mortality. Among people with AHD and co-infected with TB and histoplasmosis, therapy against all pathogens should be closely monitored in terms of levels, resistance, drug interactions and adherence to ensure the clinical success of these patients.

## Supplementary Material

trad104_Supplemental_File

## Data Availability

The data will be shared upon reasonable request to the corresponding author.
