# Supplementary material for: Clinical characteristics, diagnosis, treatment and outcomes of patients living with HIV and co-infected with tuberculosis and histoplasmosis: a 5-y retrospective case series
Source: Trans R Soc Trop Med Hyg. 2024 Jan 27;118(6):391–8. doi: 10.1093/trstmh/trad104 (PMC11149374; doi:10.1093/trstmh/trad104)
Supplement: trad104_Supplemental_File [file trad104_supplemental_file.docx]

**SUPPLEMENTARY MATERIAL**

**Table S1.** Individual histoplasmosis and tuberculosis management of the patient and six-month survival status.

**Table S1.** Individual histoplasmosis and tuberculosis management of the patient and six-month survival status.

| **ID** | **Baseline evaluation and clinical features** | | | | | | **Histoplasmosis treatment-induction therapy and maintenance therapy (MT)** | | | | | | **Tuberculosis treatment and completion of phases** | | | | **Follow-up** | |
| --- | --- | --- | --- | --- | --- | --- | --- | --- | --- | --- | --- | --- | --- | --- | --- | --- | --- | --- |
|  | **Age (years)** | **Sex†** | **CD4- cells/uL** | **Type Histo†** | **Type TB¥** | **Days from Dx TB to Dx Histo** | **Days from DX histo to histo treatment** | **Induction therapy¶** | **Doses** | **MT1** | **MT2** | **MT3** | **Days from diagnosis to TB treatment** | **Received** | **Initiation phase** | **Continuation phase** | **Days** | **Survival status §** |
| 1 | 25 | M | 39 | PH | PTB | 0 |  | No |  |  |  |  |  | No | No |  | 13 | Early death |
| 2 | 41 | M | 19 | SD | ETB | -7 | 9 | DAmB 0.7 mg/kg/d | 2 | No |  |  | 2 | Yes | No |  | 18 | Early death |
| 3 | 27 | M | 2 | MD | ETB | -2 | 1 | DAmB 0.7 mg/kg/d | 5 | No |  |  |  | No | No |  | 6 | Early death |
| 4 | 58 | F | 129 | SD | PTB | 0 | 1 | DAmB 0.7 mg/kg/d | 10 | No |  |  | 1 | Yes | No |  | 15 | Early death |
| 5 | 43 | F | 33 | SD | PTB | 1 |  | No |  |  |  |  | 1 | Yes | No |  | 21 | Early death |
| 6 | 37 | M | 20 | SD | PTB | 4 | 0 | DAmB 0.7 mg/kg/d | 14 | No |  |  | 0 | Yes | No |  | 166 | Late death |
| 7 | 30 | M | 2 | SD | ETB | 0 | 3 | DAmB 0.7 mg/kg/d followed by DAmB 0.7 mg/kg every 48 hours | 14 + 7 | ITZ 200 mg TID |  |  | 7 | Yes | Yes | No | 119 | Late death |
| 8 | 38 | M | 5 | SD | ETB | 101 |  | No |  |  |  |  | -2 | Yes | Yes |  | 136 | Late death |
| 9 | 44 | M | 18 | PH | PTB | 77 | 9 | ITZ 200 mg TID | 9 | ITZ 200 mg TID |  |  | 0 | Yes | Yes | No | 104 | Late death |
| 10 | 43 | M | 73 | SD | ETB | 15 | 0 | DAmB 0.7 mg/kg/d | 14 | ITZ 200 mg TID |  |  | 1 | Yes | No |  | 109 | Late death |
| 11 | 39 | M | 18 | SD | ETB | -20 | 5 | DAmB 0.7 mg/kg/d | 14 | ITZ 200 mg TID | ITZ 200 mg BID | ITZ 200 mg TID | 8 | Yes | No |  | 111 | Late death |
| 12 | 37 | M | 50 | SD | PTB | 0 | 3 | DAmB 0.7 mg/kg/d | 21 | ITZ 200 mg TID |  |  | 3 | Yes | Yes | No | 146 | Late death |
| 13 | 20 | M | 4 | SD | ETB | -2 | 1 | DAmB 0.7 mg/kg/d | 14 | ITZ 200 mg TID |  |  | 2 | Yes | Yes | Yes | 205 | Survivor |
| 14 | 40 | M | 82 | SD | PTB | 0 | 6 | DAmB 0.7 mg/kg/d | 14 | ITZ 200 mg BID | ITZ 200 mg TID | ITZ 200 mg BID | 6 | Yes | Yes | Yes | 218 | Survivor |
| 15 | 41 | F | 45 | SD | ETB | 4 | 3 | DAmB 0.7 mg/kg/d | 14 | ITZ 200 mg TID | ITZ 200 mg BID |  | 6 | Yes | Yes | Yes | 236 | Survivor |
| 16 | 26 | M | 1 | SD | ETB | 0 | 2 | DAmB 0.7 mg/kg/d | 14 | ITZ 200 mg TID | ITZ 200 mg BID |  | 4 | Yes | Yes | Yes | 191 | Survivor |
| 17 | 46 | M | 28 | SD | ETB | -46 | 1 | DAmB 0.7 mg/kg/d | 14 | ITZ 200 mg BID |  |  | 2 | Yes | Yes | Yes | 210 | Survivor |
| 18 | 31 | M | 60 | MD | PTB | 0 | 7 | ITZ 200 mg TID | 9 | ITZ 200 mg TID | ITZ 200 mg BID |  | 18 | Yes | Yes | Yes | 175 | Survivor |
| 19 | 28 | M | 47 | SD | PTB | 0 | 4 | DAmB 0.7 mg/kg/d + liposomal amphotericin B 1 mg/kg/d | 7 + 12 | ITZ 200 mg TID | ITZ 200 mg BID |  | 10 | Yes | Yes | Yes | 228 | Survivor |
| 20 | 23 | M | 20 | SD | PTB | 0 | 5 | DAmB 0.7 mg/kg/d | 14 | ITZ 200 mg TID | ITZ 200 mg QD |  | 11 | Yes | Yes | Yes | 167 | Survivor |
| 21 | 26 | M | 8 | SD | ETB | -3 | 3 | DAmB 0.7 mg/kg/d | 13 | ITZ 200 mg TID | ITZ 200 mg BID |  | 5 | Yes | Yes | Yes | 196 | Survivor |

† M= masculine, F=feminine; ^†^ Type Histo=Histoplamosis, DS= severe disseminated histoplasmosis, DM=mild to moderated disseminated histoplasmosis, PH=pulmonary histoplasmosis; ^¥^TB=tuberculosis, PTB=pulmonary tuberculosis, ETB=extrapulmonary tuberculosis; Î DX=Diagnosis, Tx=Treatment, Negative values indicate that diagnosis tuberculosis occurred first; **Ω** = Negative values indicate that treatment was provided before diagnosis; ¶ = DAmB = amphotericin B deoxycholate, ITZ=itraconazole.  **^§^**Early death: Death ≤ 30 days of medical evaluation; Late death: Death > 30 days of medical evaluation, Survivor=alive after 6 months of medical evaluation.
